# Relative efficacy of organic acids and antibiotics as growth promoters in broiler chicken

**DOI:** 10.14202/vetworld.2016.377-382

**Published:** 2016-04-14

**Authors:** Vikrant Laxman Bagal, Vinod Kumar Khatta, Bachu Singh Tewatia, Sandeep Kumar Sangwan, Subhash Shamrao Raut

**Affiliations:** 1 Department of Animal Nutrition, Lala Lajpat Rai University of Veterinary and Animal Sciences, Hisar, Haryana, India; 2 Department of Animal Genetics and Breeding, Lala Lajpat Rai University of Veterinary and Animal Sciences, Hisar, Haryana, India; 3 Department of Livestock Product and Technology, Lala Lajpat Rai University of Veterinary and Animal Sciences, Hisar, Haryana, India

**Keywords:** antibiotics, broilers, economics, growth promoters and organic acids

## Abstract

**Aim:**

The objective of this study was to evaluate the effect of organic acids as replacer to antibiotics in their various combinations on feed consumption, body weight gain, and feed conversion ratio (FCR) in broiler chicks during different phases of growth.

**Materials and Methods:**

Antibiotics and organic acids were incorporated into boiler feed in different combinations to form 10 maize based test diets (T_1_ to T_10_). Each test diet was offered to four replicates of 10 birds each constituting a total of 400 birds kept for 45 days.

**Results:**

Significantly better effect in terms of body weight gain from supplementation of 1% citric acid and 1% citric acid along with antibiotic was observed throughout the entire study, whereas the effect of tartaric acid supplementation was similar to control group. Citric acid (1%) along with antibiotic supplementation showed highest feed intake during the experimental period. Significantly better FCR was observed in groups supplemented with 1% citric acid and 1% citric acid along with antibiotic followed by antibiotic along with organic acids supplemented group.

**Conclusion:**

Growth performance of birds in terms of body weight, body weight gain, and FCR improved significantly in 1% citric acid which was significantly higher than antibiotic supplemented group. 1% citric acid can effectively replace antibiotic growth promoter (chlortetracycline) without affecting growth performance of birds.

## Introduction

An Indian organized poultry industry is a fastest growing sector with annual growth rate of around 810% over the last decade with 2.47 million tons of broiler meat [1]. Knowledge of poultry nutrition, disease prevention, and scientific management practices and genetically improved breeds have significantly increased production in the poultry sector. The total poultry population in India is 729.2 million [2]. India occupies the third position for producers of hen eggs while in chicken meat production India occupies the sixth position in the world [3]. Presently, annual per capita availability of eggs and meat in India is 49 and 2.5 kg, respectively. However, according to the National Institute of Nutrition recommendation per capita requirement of eggs and meat is 180 eggs and 11 kg meat/year [4].

In 1950’s, poultry farmers discovered that feeding low doses of antibiotics to their birds help birds to gain weight faster. A non-qualified person while working with antibiotics may create an environment in which bacteria can develop antibiotic resistance. Microorganisms such as *Escherichia coli* and *Klebsiella pneumoniae*, which are commensals and pathogens for humans and animals, have become increasingly resistant to third-generation cephalosporins. Moreover, in certain countries, they are also resistant to carbapenems, and therefore, susceptible only to tigecycline and colistin [5]. Resistance to antibiotics has increased dramatically over the past few years and has now reached a level that places future patients in real danger. Recognizing increasing antimicrobial resistance as a major public health issue, the World Health Organization strongly recommended banning or restricting non-therapeutic use of antibiotics in veterinary practices [6]. In response to this apparent threat, European Union banned the use of antibiotics at sub therapeutic level as a growth promoter or for disease prevention. In the developing countries, for preventing metabolic stress or subclinical infections in broiler chicks, the use of antibiotics needs to be replaced by suitable growth promoters. In search of an efficient alternative to antibiotic growth promoters, a number of agents are being tried.

Organic acids associated with specific antimicrobial activity are short chain acids (C1-C7) and are simple monocarboxylic acids such as formic, acetic, propionic, butyric acid, or carboxylic acid bearing hydroxyl group such as citric, tartaric, lactic, and malic acid. Lactic acid is most effective against bacteria. The use of organic acids, such as citric, formic, fumaric, lactic, and propionic acid, has shown improved digestibility, improved mineral absorption thus beneficial effect in feed efficiency in pigs [7]. Their addition generally lowers the pH, increase proteolysis, nutrient digestibility, and preservation of feed nutrients which improves the overall performance of the birds.

In addition, the acid anion forms complexes with mineral elements and facilitates their uptake from the gut. Organic acids improve nutrient metabolizability in broilers and have been found to be one of the most promising alternatives to antibiotic growth promoters and prophylactics against enteric diseases [8]. Keeping in view the aforesaid facts, a comparative study was designed to put on record the effect of organic acids and antibiotics in their various combinations as growth promoters in broiler chicken.

## Materials and Methods

### Ethical approval

The research work was carried out after approval of the Institutional Animal Ethics Committee, College of Veterinary College, Lala Lajpat Rai University of Veterinary and Animal Sciences (LUVAS), Hisar, for a period of 45 days starting from 8^th^ December 2012 at poultry shed of the university, with due care to minimize pain and discomfort to the birds.

### Formulation of experimental diets

About 10 maize based test diets for each growth period (starter and finisher ration) for broilers were formulated as per BIS, 1992 [9] to meet out metabolizable energy (ME), crude protein (CP), and limiting amino acids requirements having different combinations of antibiotics and organic acids (T_1_ to T_10_). All feed ingredients (maize, soybean meal, fish meal, and rice polish), additives (mineral mixture, vitamins, coccidiostat, choline chloride, lysine, and DL-methionine), and growth promoters (antibiotic and organic acids preparation) were procured in one lot before the start of the experiment. Feed ingredients used for diet formulations were analyzed for proximate nutrients [10]. Analysis report as per methods of analysis of AOAC [10] of ingredients used in feed formulation is given in Table-1. The CP level was 23% in starter and 20% in finisher diets while the ME content was 2800 and 2900 kcal/kg in the respective groups. The formulation used for making ration for starter and finisher growth period is given in Table-2. Chemical composition (% dry matter basis) of experimental diets in different growth phases of broiler chicks is given in Table-3. The level of growth promoters used in each test diet is given in Table-4.

**Table-1:** Chemical composition of feed ingredients.

Ingredients	CP (%)	EE (%)	CF (%)	TA (%)	Lysine* (%)	Methionine* (%)	ME* (Kcal/kg)	Cost (Rs./Qtls.)
Maize	9.1	3.44	2.44	2.25	0.18	0.15	3300	1200
Soybean meal	45.2	3.16	3.93	8.47	2.57	0.76	2230	2500
Rice polish	12.0	12.2	4.10	14.94	-	-	2937	1000
Fish meal	45.0	13.5	1.79	39.62	1.42	1.42	2600	2600

* Calculated values (Singh and Panda [26]). CP=Crude protein, EE=Ether extract, CF=Crude fiber, TA=Total ash, ME=Metabolizable energy

**Table:** 

	0-4 weeks	4-6 weeks
Name of ingredients (kg/100 kg feed)		
Maize	49.11	57.58
Soybean meal	28.38	20.12
Rice polish	10.0	10.0
Fish meal	10.0	10.0
Mineral mixture	2.5	2.5
Feed additives (g/100 kg feed)		
Spectromix	10	10
Spectro BE	20	20
Veldot	50	50
Choline chloride	50	50
Lysine	50	50
DL-methionine	150	150
CTC-150 (g/kg)	33.5	33.5
Citric acid (%)	0.5, 1	0.5, 1
Tartaric acid (%)	0.5, 1	0.5, 1

Spectromix: Powder (Ranbaxy Animal Health, New Delhi-65). Each gram contained Vitamin A - 82,500 IU, Vitamin D3 - 12,000 IU, Vitamin B2 - 50 mg and Vitamin K – 10 mg; mixing rate: 10 g/100 kg of feed. Spectro BE: Powder (Ranbaxy Animal Health, New Delhi-65). Each gram contained Vitamin B1-8 mg, Vitamin B6 - 16 mg, Vitamin B12 - 80 mg, niacin - 120 mg, calcium pantothenate – 80 mg, Vitamin E - 160 mg, lysine hydrochloride - 10 mg, DL-methionine - 10 mg and calcium – 260 mg; mixing rate: 20 g/100 kg of feed. Veldot: Venkys-Dinitro-O-Toluamide (Coccidiostat); mixing rate: 50 g/100 kg feed. Choline chloride: Contain 60% choline; mixing rate: 50 g/100 kg of feed. Lysine: Contained 98% lysine; mixing rate: 50 g/100 kg of feed. DL-methionine: Contained 98% methionine; mixing rate: 150 g/100 kg of feed

**Table-3:** Chemical composition (% DM basis) of experimental diets in different growth phases of broiler chicks.

Attributes	0-28 days	29-45 days
Moisture	11.47	11.11
DM	88.55	88.89
CP	22.91	20.63
EE	5.00	5.14
CF	4.33	5.00
Ash	9.00	9.15
NFE	47.33	48.97

DM=Dry matter, CP=Crude protein, EE=Ether extract, CF=Crude fiber, NFE=Nitrogen free extract

**Table-4:** Different dietary treatments.

T_1_	Control diet
T_2_	Basal diet+antibiotic (0.0335% chlortetracycline)
T_3_	Basal diet+antibiotic (0.0335% chlortetracycline)+ 0.5% citric acid
T_4_	Basal diet+antibiotic (0.0335% chlortetracycline)+ 1% citric acid
T_5_	Basal diet+0.5% citric acid
T_6_	Basal diet+1% citric acid
T_7_	Basal diet+antibiotic (0.0335% chlortetracycline)+ 0.5% tartaric acid
T_8_	Basal diet+antibiotic (0.0335% chlortetracycline)+ 1% tartaric acid
T_9_	Basal diet+0.5% tartaric acid
T_10_	Basal diet+1% tartaric acid

### Design of experimental work

The experiment was planned following completely randomized design at uniform and standard management practices. 400, day-old broiler chicks of Cobb strain were procured from a local commercial hatchery. The chicks were wing banded, weighed, and randomly distributed into 40 subgroups (10 dietary treatments with four replicates per treatment) of 10 birds in each. The experiment lasted for 45 days, which was conveniently divided into three 15 day interval periods. Care and management of birds were done as per standard managemental practices with *ad libitum* feed and water.

### Feed consumption

Feed consumption per bird was calculated after every 15 days by dividing the difference of total amount of feed offered and the residual amount of feed for each replicate by a number of birds in that replicate.

### Body weight gain

The birds were weighed individually at 15 days interval. Body weight gain of each bird was calculated by subtracting recorded body weights at different intervals.

### Feed conversion ratio (FCR)

FCR for each replicate was calculated by dividing the total feed consumed by that replicate and total body gain by all the birds in that replicate.

### Statistical analysis

Data on body weight gain, feed consumption, and FCR were subjected to one-way ANOVA statistical analysis as per the standard procedure recommended by Snedecor and Cochran [11]. Observations due to different treatments were compared for differing significance at 1% and 5% level using modified Duncan’s multiple range test [12]

## Results

Observations of various traits under study were statically analyzed to find out the comparative effects of various test diets on broiler birds.

### Feed consumption

The results about feed intake (g/bird) during different growth periods under different dietary treatments are presented in Table-5. During overall growth period (0-45 days) feed intake under different dietary treatments varied from 4222.2 g (T_1_) to 4862.3g (T_4_). Lower feed intake was observed in control followed by control +0.5% tartaric acid, control +0.5% citric acid, and control +1% tartaric acid which was comparable within these groups. The dietary supplementation of antibiotic growth promoter alone or in combination with organic acids in treatments (T_2_, T_3_, T_6_, T_7_, and T_8_) showed feed intake comparable within these treatments but differed significantly (p<0.05) higher than control and organic acids combinations (T_1_, T_5_, T_9_, and T_10_). Feed consumption did not differ significantly among control and organic acid supplemented groups.

**Table-5:** Feed intakes (g/bird) during different growth periods under different dietary treatments.

Treatments	0-15 days	15-30 days	30-45 days	0-45 days
T_1_	868.7^a^±04.7	1535.9^a^±05.4	2017.7^a^±08.2	4222.2^a^±08.8
T_2_	941.4^b^±04.5	1641.4^b^±07.0	2139.1^b^±13.8	4701.6^b^±12.2
T_3_	915.6^b^±10.0	1638.6^b^±10.8	2152.4^b^±19.7	4706.6^b^±22.2
T_4_	949.9^b^±06.2	1672.8^c^±03.9	2239.6^c^±09.4	4862.3^c^±11.8
T_5_	876.7^a^±20.9	1554.4^a^±21.8	2030.5^a^±25.2	4465.1^a^±16.7
T_6_	914.1^b^±08.8	1636.4^b^±16.8	2130.0^b^±23.7	4751.6^b^±21.4
T_7_	921.6^b^±10.8	1645.0^b^±09.5	2134.5^b^±08.9	4762.6^b^±13.8
T_8_	999.0^b^±13.1	1611.7^b^±07.3	2147.2^b^±23.5	4678.0^b^±26.1
T_9_	862.2^a^±10.1	1569.7^a^±07.5	2047.2^a^±16.5	4449.1^a^±18.3
T_10_	876.7^a^±36.8	1542.9^a^±10.3	2083.3^a^±09.7	4479.9^a^±18.5

Means bearing different superscripts in a column differ significantly (p<0.05)

Adil *et al*. [13] also found similar results, i.e. non-significant decrease in feed consumption due to 2% butyric acid, 3% butyric acid, 2% fumaric acid, 3% fumaric acid, 2% lactic acid, and 3% lactic acid supplementation. However, in contrast to present finding Pirgozliev *et al*. [14] observed decreased feed intake in organic acid supplemented group as compared to control. Shahin *et al*. [15] observed that 1% citric acid level in feed reduced feed intake significantly (p<0.05) as compared to 0.2% and 0.4% levels. Boling *et al*. [16] found that higher level of citric acid in diet lowered feed intake in birds, which might be due to shock decrease in palatability of acidified diets during the first stage. However, during the second stage, the birds accustomed the dietary acidification.

### Weight gain

The results pertaining body weight gain during different growth periods of comparative efficacy of organic acids and antibiotics in different dietary treatments as a growth promoter in broiler chick are presented in Table-6. Average body weight gain at 15^th^ days was the highest in birds supplemented with 1% citric acid along with antibiotic growth promoter (T_4_) and 1% citric acid (T_6_) which was significantly different from the control, 0.5% citric acid and 0.5% and 1% tartaric acid supplemented treatments (T_1_, T_5_, T_9_, and T_10_). Addition of antibiotic growth promoter to basal diet increased average body weight gain in the presence or absence of acidifier which was significantly (p<0.05) higher than the control followed by 0.5% citric acid and 0.5% and 1% tartaric acid supplemented treatments (T_1_, T_5_, T_9_, and T_10_). Similar results were also obtained during 15-30 days and 30-45 days growth period. At 45 days, 1% citric acid and 1% citric acid along with antibiotic growth promoter had comparable average body weight gain, which was higher than all other dietary treatment.

**Table-6:** Average weight gain (g/bird) during different growth periods under different dietary treatments.

Treatments	0-15 days	15-30 days	30-45 days	0-45 days
T_1_	410.4^a^±05.6	798.2^a^±18.5	1027.0^a^±15.8	2235.6^a^±30.4
T_2_	459.4^b^±00.6	852.1^b^±19.4	1092.6^b^±14.8	2404.4^b^±18.1
T_3_	459.9^b^±05.1	875.9^b^±10.6	1093.4^b^±13.6	2485.5^b^±31.6
T_4_	511.6^c^±06.7	932.2^c^±23.3	1226.9^c^±18.3	2614.4^c^±16.2
T_5_	466.8^a^±05.6	773.9^a^±02.7	1045.0^a^±08.8	2225.7^a^±12.9
T_6_	495.7^c^±10.2	885.7^c^±24.9	1171.0^c^±13.0	2553.3^c^±17.4
T_7_	446.2^b^±03.9	858.0^b^±14.5	1098.5^b^±24.2	2402.7^b^±24.3
T_8_	447.9^b^±03.7	846.5^b^±09.9	1088.5^b^±20.8	2383.1^b^±27.0
T_9_	409.7^a^±01.5	774.1^a^±02.3	1027.7^a^±03.7	2211.6^a^±02.0
T_10_	413.0^a^±04.4	837.5^a^±09.3	1002.8^a^±28.5	2193.9^a^±19.6

Means bearing different superscripts in a column differ significantly (p<0.05)

Various researchers also reported similar improved weight gain with dietary supplementation of organic acid [17,18], acidifiers [19,20], and citric acid [15,21-23]. Improvement in the body weight gain recorded in the present study could be attributed to the direct antimicrobial effect of organic acids which might have resulted in inhibition of intestinal bacteria leading to the reduced bacterial competition with the host for available nutrients and reduction in the level of toxic bacterial metabolites as a result of lessened bacterial fermentation resulting in the improvement of protein and energy digestibility, thereby improving the weight gain of broiler chicken [13]. Antibiotic growth promoters reduce subclinical infection; growth depressing metabolites reduce microbial utilization of nutrients and increase absorption of nutrients by reducing the thickness of intestinal mucosa [24]. Organic acid supplementation reduces the growth of many pathogenic and non-pathogenic intestinal bacteria due to reduction in intestinal colonization and infectious process. Organic acid also improves villus height and function of secretion, digestion, and absorption of nutrients lead to overall increase in average weight gain of birds [8]. Therefore, the action of antibiotic and citric acid are a synergistic action which produces a better response when fed in combination.

### FCR

Results pertaining to the FCR of birds fed with different antibiotic and organic acid (as growth promoter) dietary treated feeds during different growth periods are presented in Table-7. During 0-15 days of the growth period, FCR under different dietary treatments ranged from 1.82 (T_6_) to 2.14 (T_5_). Supplementation of tartaric acid at 0.5% and 1% concentrations and citric acid at 0.5% concentration in basal diet did not alter FCR significantly than that of control treatment. T_2_ (control + antibiotic), T_3_ (control + antibiotic + 0.5% citric acid), T_7_ (control + antibiotic + 0.5% tartaric acid), and T_8_ (control + antibiotic + 1% tartaric acid) in which antibiotics are supplemented alone or in combination with citric acid (0.5%) and tartaric acid (0.5% and 1%) showed improved FCR as compared to T_1_ (control), T_5_ (control +0.5% citric acid), T_9_ (control +0.5% tartaric acid), and T_10_ (control + 1% tartaric acid). The best FCR was recorded in treatment T_4_ (control + antibiotic +1% citric acid) and T_6_ (control + 1% citric acid) than all other dietary treatments. Similar result was obtained during 15-30 days and 30-45 days growth period. At 45 days, 1% citric acid and 1% citric acid along with antibiotics had comparable FCR, which was significantly (p<0.05) better than all other dietary treatments.

**Table-7:** FCR during different growth periods under dietary treatments.

Treatments	0-15 days	15-30 days	30-45 days	0-45 days
T_1_	2.11^d^±0.03	1.92^bc^±0.04	1.96^bc^±0.03	1.97^ef^±0.03
T_2_	2.00^bc^±0.02	1.92^bc^±0.04	1.95^bc^±0.03	1.95^cd^±0.02
T_3_	1.99^bc^±0.03	1.76^a^±0.04	1.95^bc^±0.04	1.89^ab^±0.03
T_4_	1.85^a^±0.02	1.91^bc^±0.02	1.82^abc^±0.02	1.86^a^±0.01
T_5_	2.14^d^±0.03	2.00^cd^±0.03	1.94^bc^±0.03	2.00^ef^±0.02
T_6_	1.82^a^±0.02	1.80^b^±0.03	1.85^ab^±0.03	1.83^aa^±0.01
T_7_	2.06^c^±0.02	1.91^bc^±0.04	1.94^bc^±0.05	1.96^cd^±0.02
T_8_	2.05^c^±0.02	1.90^bc^±0.01	1.97^bc^±0.04	1.96^cd^±0.02
T_9_	2.10^d^±0.01	2.02^d^±0.01	1.99^bc^±0.01	2.02^ef^±0.09
T_10_	2.12^d^±0.05	1.98^bc^±0.02	2.05^c^±0.06	2.04^ef^±0.03

Means bearing different superscripts in a column differ significantly (p<0.05). FCR=Feed conversion ratio

Adil *et al*. [13] and Boling *et al*. [16] also reported improved FCR with supplementation of organic acids. Ghazalah *et al*. [21] found that citric acid at 2% level and 0.75% acetic acid achieved significant improvement in FCR as compared to control. Das *et al*. [25] also reported improved FCR in broiler in various citric acid, CP, and energy combinations. In this study, FCR of control and acidifier supplemented treatments was comparable except citric acid 1% supplemented group which is contrary to the findings of Fattah *et al*. [23], who reported that FCR significantly improved with any type and level of tested organic acid as compared to unsupplemented groups. The FCR of acidifier along with antibiotic supplemented treatment was comparable except antibiotic supplemented with citric acid 1% this may be due to the effect of antibiotic as the growth promoter. FCR of this treatment was found significantly better than control and acidifier group. The improvement in FCR could be possibly due to better utilization of nutrients resulting in increased body weight gain in the birds fed organic acids in the diet.

## Conclusion

Growth performance of birds in terms of body weight, body weight gain, and FCR improved significantly in 1% citric acid which was significantly (p<0.05) higher than antibiotic supplemented group. It was inferred that 1% citric acid can effectively replace antibiotic growth promoter (chlortetracycline) without affecting the performance of birds.

## Authors’ Contributions

The research was done by VLB as a part of his master’s degree program. VKK and BST designed the study and supervised the research. VLB, SKS, and SSR worked and collaborated in the lab work. VKK and BST analyzed data collected during research. VLB, SKS, and SSR compiled results as well as the manuscript. All authors read and approved the final manuscript.
